# The Benefits of Banded over Non-banded Roux-en-Y Gastric Bypass in Patients with Morbid Obesity: a Multi-center Study

**DOI:** 10.1007/s11695-022-06024-w

**Published:** 2022-04-02

**Authors:** Marijn T. F. Jense, Inge H. Palm-Meinders, Rochelle Sigterman-Nelissen, Evert-Jan G. Boerma, Ronald S. L. Liem, Dingeman J. Swank, Jan Willem M. Greve

**Affiliations:** 1 Bariatric Surgery, Zuyderland Medical Center, Henri Dunantstraat 5, 6419 PC Heerlen, Netherlands; 2 Dutch Obesity Clinic South, John F. Kennedylaan 301, 6419 XZ Heerlen, Netherlands; 3 Dutch Obesity Clinic West, The Hague, Netherlands; 4 Maastricht University Medical Center, Maastricht, Netherlands

**Keywords:** Bariatric surgery, Roux-en-Y gastric bypass, Silicone banding, Gastric bypass, Banded gastric bypass, Obesity, Weight loss

## Abstract

**Background:**

Roux-en-Y gastric bypass (RYGB) has proven to be an effective treatment for obesity with excellent long-term results, even though weight regain can occur. A method to improve the results of RYGB and minimize chance of weight regain is banded RYGB. Better sustained weight loss is also related to higher remission of comorbidities. The aim of this study was to evaluate the effect of banded and non-banded RYGB on long-term weight loss results and comorbidities.

**Method:**

A retrospective comparative data study was performed. Patients who underwent a primary RYGB between July 2013 and December 2014 and followed a 5-year follow-up program in the Dutch Obesity Clinic were included. Comorbidities were assessed during screening and follow-up.

**Results:**

The study included 375 patients with mean weight and body mass index (BMI) of 128.9 (± 21.2) kg and 44.50 (± 5.72) kg/m^2^. Of this group, 184 patients underwent RYGB and 191 banded RYGB. During follow-up (3 months, 1–5 years) % Total Weight Loss (%TWL) was superior in the banded group (32.6% vs 27.6% at 5 years post-operative, *p* < 0.001). Complication rates in both groups were similar. Comorbidity improvement or remission did not significantly differ between the two groups (*p* = 0.14–1.00). After 5 years of follow-up, 79 patients (20.5%) were lost to follow-up.

**Conclusion:**

Banded RYGB does show superior weight loss compared to non-banded RYGB. No difference in effect on comorbidity improvement or remission was observed. Since complication rates are similar, while weight loss is significantly greater, we recommend performing banded RYGB over non-banded RYGB.

**Graphical abstract:**

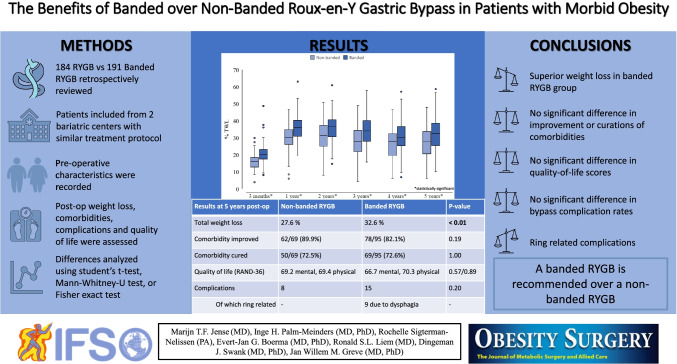

## Introduction

Achieving successful long-term weight loss after bariatric surgery remains to be a major challenge. One of the most effective treatments, when considering complication rate and weight loss combined, with the best long-term results is Roux-en-Y gastric bypass (RYGB) [1, 2]. However, even in patients who have undergone RYGB, weight regain tends to occur [3].

A possible method to improve the results of RYGB and minimize the chance of weight regain is to perform a banded RYGB. Although results are promising, not everyone is convinced of the use of a silicon ring. Pouch outlet obstruction caused by band slippage, gastroesophageal reflux disease, and dysphagia are some of the most described negative side effects of a banded procedure [4, 5].

A banded procedure has several benefits. Multiple studies showed an improvement of excess weight loss (EWL) of 15 to 20% up to 9 years post-surgery [6–8]. Furthermore, weight regain 5 years after a banded procedure decreased by more than 10% compared to non-banded procedures [9]. Besides the fact that weight loss in itself is beneficial, it is also associated with comorbidity reduction and remission. Although studies have shown benefits of a banded procedure, some have either small study populations or a relatively short study period with a maximum of 2 years [5].

The aim of this study was to determine whether a banded RYGB results in higher long-term weight loss compared to a non-banded RYGB 5 years post-operatively. As secondary objectives, this study aimed to determine the effect of a banded RYGB on the following comorbidities: type 2 diabetes, hypertension, Obstructive Sleep Apnea Syndrome (OSAS), and dyslipidemia, and on the quality of life.

## Method

### Study Design

Data was collected of patients who underwent a primary laparoscopic banded RYGB operation performed between July 2013 and December 2014 in Zuyderland Medical Center or a primary laparoscopic RYGB at the Haaglanden Medical Center or at Groene Hart Hospital. Both groups underwent the same intensive multidisciplinary group treatment for 5 years at the Dutch Obesity Clinic (DOC). For this retrospective data study, local approval was obtained from the ethics committee in accordance with the ethical standards as laid down in the 2013 Declaration of Helsinki.

### Participant and Eligibility Criteria

Patients were included if they underwent a primary laparoscopic RYGB in one of the selected centers between July 2013 and December 2014. All included patients met IFSO criteria. All operations were performed laparoscopically. In both groups, pouches were created around a 40 charrier nasogastric tube and had a length of 6–8 cm. The biliopancreatic limbs had a length of 60 cm, the alimentary limb had a length of 120 cm. The gastrojejunostomy and the jejunojejunostomy were created using a linear stapler and hand-sewn closure of the remaining opening. In all centers, the surgical technique was similar. Only in the banded group, a silicone ring was placed 1–2 cm above the gastrojejunostomy. The silicon ring was created using a medical grade ventriculoperitoneal drain (8 Fr), which was approved in hydrocephalus management and therefore approved for long-term implantation. The tube size was standardized depending on gender, 6.5 cm for females and 7.0 cm for males. Using two non-resorbable stitches, the band was closed around the pouch. All rings were placed loosely around the pouch and did not cause any restriction; a 5-mm grasper should easily fit between the band and the gastric wall of the pouch.

The patients followed the standard 5-year extensive follow-up treatment of the DOC by a multidisciplinary team, consisting of a dietician, psychologist, exercise therapist, medical doctor, and a surgeon.

Patients were excluded for the following three reasons: if they received a Roux-en-Y gastric bypass as conversional surgery, if they received an adjustable band, or if they followed an individual treatment instead of the standard group treatment at the DOC.

Because of the retrospective nature of this article, informed consent was not obtained for this specific study.

### Loss to Follow-Up

Since the standard follow-up period of the DOC consists of 5 years, the majority of patients had their weight and comorbidities examined at the follow-up appointments at the clinic. Missing data at 5-year follow-up was recorded by means of a questionnaire via email. Hereafter, patients were contacted via telephone, if this email and reminders remained unanswered.

### Comorbidities

During screening and at all follow-up moments, all patients were asked to report about the following doctor diagnosed comorbidities: type 2 diabetes, hypertension, Obstructive Sleep Apnea Syndrome (OSAS), and dyslipidemia. The comorbidities, and remission and improvement, were defined using the ASMBS standard article [10]. If patients did not show up for the 5-year follow-up consult, they were sent a questionnaire through email including questions about their comorbidities.

### Complications

All complications were registered via a national database. In this database, the occurrence of the complication was registered as perioperative, < 30 days without readmission, < 30 days with readmission, > 30 days without readmission, or > 30 days with readmission. All complications were graded using the Clavien Dindo classification.

### Quality of Life

All patients filled out the RAND-36 questionnaire at the following timepoints: start of treatment, 15, 24, 36, 48, and 60 months post-operative. The RAND-36 questionnaire consists of different scales concerning either mental of physical wellbeing as the main category. These categories consist of subcategories such as social functioning, emotional problems, pain, and general wellbeing [11]. The scores of the RAND-36 questionnaire in this study were displayed for the main categories only.

### Experience

After the 5-year follow-up period, a questionnaire was sent to the patients with the following questions: which comorbidities (hypertension, diabetes, dyslipidemia, OSAS) are still present?; what is your current weight?; would you undergo treatment again?; and if a band was placed, is the band still in place or did you undergo a removal or replacement operation?

### Statistical analysis

Statistical analysis was performed using the IBM SPSS Statistics for Windows, version 26, Armonk NY. Firstly, all results were tested for normality using the Kolmogorov–Smirnov test. Differences were tested for significance using a two-tailed Student’s *t*-test or a Mann–Whitney-U test, and a chi-square test or Fishers exact test. A *p*-value of less than 0.05 was regarded as statistically significant.

## Results

Out of the 375 evaluated patients, 184 received a non-banded RYGB (49.1%) and 191 a banded RYGB (50.9%). The non-banded group comprised of 147 female patients (79.9%). The group had a mean age of 43 years (± 10) and a mean preoperative BMI of 44.7 kg/m^2^ (± 5.5). The banded group comprised of 136 female patients (71.2%). The group had a mean age of 43 years (± 11) and a mean preoperative BMI of 44.3 kg/m^2^ (± 5.9). As shown in Appendix Table 1, there were no significant differences in comorbidities at baseline except for a higher prevalence of OSAS in the banded group (*p* < 0.001).

### Loss to Follow-Up

Per time point, different percentages of patients were lost to follow-up, ranging from 19.5 to 58.4% as presented below in the figures and tables. After the 5-year follow-up, 79 patients (20.5%), 49 from the non-banded, and 30 from the banded group could not be reached. The number of loss to follow-up did not significantly differ at any timepoint except for 5-year follow-up (*p* = 0.01). Of the 79 patients, two patients had died during the follow-up period. Their deaths were not related to the bariatric procedure.

### Weight Loss

The weight loss results, at 3 months, and annually up to 5 years postoperatively, differed between the two study groups. Appendix Fig. 1 and Appendix Table 2 present the differences between the banded and non-banded group in percentage TWL at all follow-up points. At all follow-up time points, the percentage of TWL was significantly higher in the banded group compared to the non-banded group. For the non-banded group, the mean percentage TWL was 16.0, 30.4, 31.4, 28.6, 27.4, and 27.6 for 3 months, and yearly up to 5 years, respectively. For the banded group, the mean %TWL was 20.5, 35.6, 35.6, 34.0, 31.0, and 32.6 for 3 months, and annually up to 5 years, respectively. This resulted in a mean difference of 5.0%TWL at 5 years after surgery, *p* < 0.001.

For all patients %EWL was also calculated. In Appendix Fig. 2 and Appendix Table 3, the differences in %EWL at all follow-up points are shown. At all follow-up points, except for 4 years post-operative, the differences were statistically significant in favor of the banded RYGB.

### Comorbidities

During screening and at 5 years post-operative, there were no significant differences between the two study groups in either improvement or complete disease remission of any of the following comorbidities: hypertension, diabetes mellitus, OSAS and dyslipidemia (Appendix Table 4 and Appendix Table 5).

### Complications

A total of 23 patients with complications were registered in the study group of 375 patients. Of these 23 patients, 8 were from the non-banded group and 15 from the banded group. The complications consisted of internal herniation, metabolic disorders, and one intestinal lesion. Of the 8 patients from the non-banded group, 7 received reinterventions; 6 laparoscopically and 1 endoscopically. Two of the 8 complications in the non-banded group occurred within 30 days post-operatively. The other 6 complications occurred after 30 days post-operatively. All patients from the non-banded group with complications were readmitted. Of the 15 patients from the banded group, 11 received reinterventions; 10 laparoscopically and 1 open. All complications in the banded group occurred after 30 days post-operatively. Two of the patients with complications had to be readmitted. The complication rates did not significantly differ between the two study groups (*p* = 0.20).

### Ring-Related Complications

Seven patients (4.3%) had their ring removed because of complaints and two patients (1.2%) underwent a silicone ring replacement operation. All 9 patients who underwent re-operation suffered from dysphagia, which caused two patient to become anorexic. After re-operation, all participants, including the two patients receiving replacement operation, no longer suffered from dysphagia. No band erosion occurred in this study population.

### Quality of Life

The RAND-36 score pre-operatively in the non-banded group had a mean score of 66.5 for mental health and 54.6 for physical health. After 5 years, the mean score was 69.2 for mental health and 69.4 for physical health. In the banded group, the mean score was 67.0 for mental health and 48.8 for physical health. After 5 years, the mean score was 66.7 for mental health and 70.3 for physical health. There were no significant differences between the two study groups at either time point.

### Experience

After the 5-year follow-up period, the questionnaire as mentioned in the method section was sent to the patients. Of all 375 patients, 98/184 (53.3%) of the non-banded group and 154/191 (80.6%) of the banded group responded. The results of the questionnaire presented a similar number of patients who would participate again in both groups, 86.7% of the non-banded and 82.4% of the banded group (*p* = 0.63). There were no significant differences in willingness to undergo treatment again.

## Discussion

This study shows that a banded RYGB results in significantly higher long-term weight loss compared to a non-banded RYGB 5 years post-operatively. These results are in accordance with numerous other studies with a follow-up period of maximal 2 years [4]. Additionally, studies with a follow-up period of 5 to 10 years presented a superior effect of the banded RYGB on weight loss as well [6, 7, 12, 13]. For example, the study of Heneghan et al. which showed a mean %EWL in the non-banded group of 62.3% compared to 81.7% in the banded group, similar results compared to the present study (65.2% in RYGB vs 77.6% in banded RYGB) [6, 7, 12, 13].

Other studies on the effect of banded versus non-banded RYGB yielded conflicting results. Moon et al. compared the banded and non-banded RYGB in a study population of 1150 patients, with 543 in the banded group vs 607 in the non-banded group. However, the ring used in their study was a biological pericardial patch, which dissolves over time. At 3 years post-operative, the mean percentage excess BMI loss was 73.3% in the banded group versus 74.8% in the non-banded group, not statistically significant different. They concluded that there is only a temporary effect of the biological ring until it is dissolved [14]. Creating a logical difference with the current study with a silicone ring.

A smaller prospective study by Bessler et al., with a study population of 90 patients, presents similar results as Moon et al. for 24 months post-operative. However, at 36 months post-operative, there was a significant difference between the banded and the non-banded group in favor of the banded group (73.4% versus 57.7%) [15].

Besides, a silicon ring one can also use an adjustable band to achieve restriction. Although positive results have been reported concerning the adjustable band in a banded RYGB, most studies show high complication rates (> 20%) when using an adjustable band in RYGB patients [16–18]. When using a silicone ring, as described in the current study, complication rates are at least 10% lower.

### Comorbidities

The main goal for weight loss is to prevent or cure obesity related comorbidities. The current study failed to show a significant difference in improvement or remission of comorbidities. A review performed by Buchwald et al., including 5 studies, showed no significant difference in comorbidity improvement or remission [6]. On the other hand, a study by Ryan et al. showed that weight loss results in reduction and remission of comorbidities. Moreover, they showed that when more weight was lost, more people had an improvement or remission of their comorbidities [19]. A possible explanation for no significant differences in the current study is the small number of patients with comorbidities.

### Complications

A commonly heard concern with a banded procedure is a higher complications rate compared to the non-banded procedure. The current study showed no significant difference in early complication rates between the two study groups. However, the current study showed 9 patients needing reoperation due to dysphagia, which is 4.7% of the banded group, compared to no dysphagia in the non-banded group. A study by Galal et al., with data from the same center as the current study, presented dysphagia in 5 out of 142 patients (3.5%) who underwent a banded procedure. These complaints were resolved by reoperation, similar to the current results [20].

Band erosion is a complication described in association with banded procedures. The current study showed no patients with band erosion. This result is in accordance with Lemmens et al. who reported no patients with band erosion, in a group of over a 1000 patients who underwent a banded bypass procedure over the last 10 years [6, 9]. Furthermore, Bessler et al. presented no band related problems, including band erosion, in a prospective study cohort of 46 patients with a banded RYGB and 44 patients with a non-banded RYGB [15]. Altogether, complication rates are not higher and band erosion is shown to be a rare complication after banded RYGB.

### Quality of Life

The quality of life did not significantly differ between the banded and the non-banded group.

Even though the banded group had a higher rate of re-operations, their willingness to undergo treatment again was similar in both groups. Similar results were demonstrated in studies performed by Figueiredo Reis et al. and Awad et al., with a significant higher incidence of food tolerance in the non-banded group but no significant difference in food quality in either group. These results show that even though there is a difference in food tolerance, the patients did not experience this as a negative effect on their quality of life [21, 22].

### Limitations of the study

A retrospective study with a long follow-up period, as the current study, can cause a higher rate of loss to follow-up. However, compared to a study by Magro et al. who had 29.1% patients at 5 years post-operatively, this study has a good proportion of 79.5% patients at 5-year follow-up [12].

## Conclusion

Banded Roux-en-Y gastric bypass demonstrates a significantly higher weight loss compared with a non-banded Roux-en-Y gastric bypass, especially in the long term. The increased weight loss of the banded intervention did not result in a difference in improvement or remission of comorbidities between the two groups. Complication rates in both study groups were similarly low. Based on these study results, we recommend performing a banded over a non-banded Roux-en-Y gastric bypass.

## Figures and Tables

**Fig. 1:**
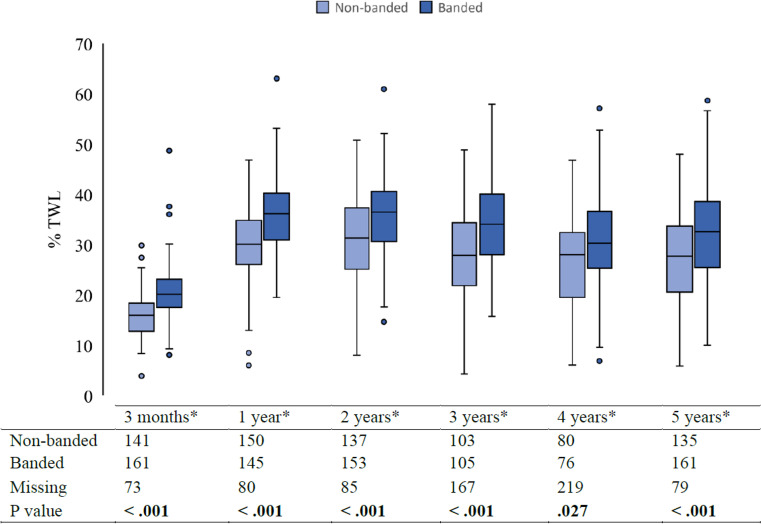
Boxplot %TWL over 5-year follow-up. *P* value calculated with Mann–Whitney U test. *significant result with a cut off point of 0.05

**Fig. 2:**
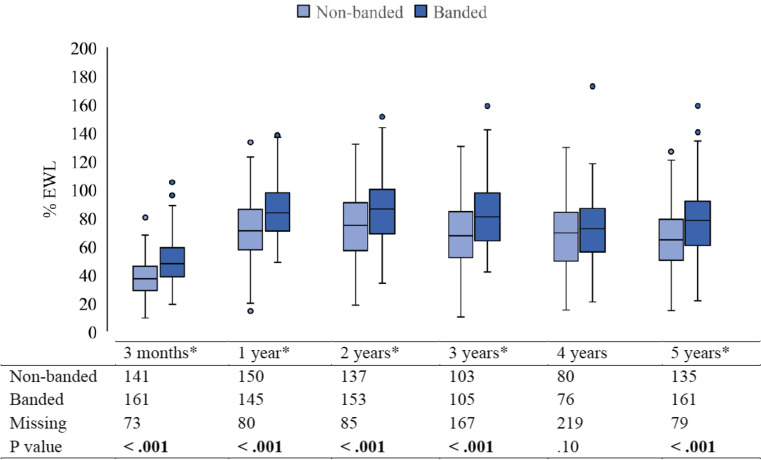
Boxplot %EWL over 5-year follow-up. *P* value calculated with Mann–Whitney U test. *significant result with a cutoff point of 0.05

## Appendix

**Table 1:** Baseline characteristics as reported during screening

Baseline characteristics		*N*	Total	*N*	Non-banded	*N*	Banded	*P* value*
Sex	*Male(%)*	92	92 (24.5)	37	37 (20.1)	55	55 (28.8)	0.055
	*Female(%)*	283	283 (75.5)	147	147 (79.9)	136	136 (71.2)	
Mean age on day of operation (SD)		375	43 (± 11)	184	43 (± 10)	191	43 (± 11)	0.90
Mean weight screening (SD)		375	128.9 (± 21.2)	184	128.5 (± 20.1)	191	129.4 (± 22.3)	0.67
Mean BMI screening (SD)		375	44.50 (± 5.72)	184	44.70 (± 5.53)	191	44.31 (± 5.90)	0.51
Hypertension (%)		372	150 (40.3)	183	72 (39.3)	189	78 (41.3)	0.75
*BP medication (%)*		372	139 (37.4)	183	66 (36.1)	189	73 (38.6)	0.67
Diabetes (%)		372	93 (25.0)	182	45 (24.6)	189	48 (25.4)	0.91
*DM medication (%)*		372	80 (21.5)	183	36 (19.7)	189	44 (23.3)	0.45
*Oral DM medication(%)*		372	74 (19.9)	183	36 (19.7)	189	38 (20.1)	1.00
*Insulin use (%)*		372	30 (8.1)	183	11 (6.0)	189	19 (10.1)	0.18
Dyslipidemia (%)		374	76 (21.6)	183	39 (21.3)	191	37 (19.4)	0.70
OSAS (%)		374	74 (21.3)	183	21 (11.6)	191	53 (27.7)	** < 0.001**
*MAS (%)*		374	21 (6.0)	182	0 (0.0)	191	21 (11.0)	** < 0.001**
*CPAP (%)*		374	41 (11.8)	182	15 (8.3)	191	26 (13.6)	0.10

**P* value calculated with Fisher exact test for binary data. For non-binary data, an unpaired *t*-test was performed. *DM*, diabetes mellitus; *OSAS*, Obstructive Sleep Apnoea Syndrome; *MAS*, mandibular advancement splint; *CPAP*, continues positive airway pressure; *SD*, standard deviation

**Table 2:** TWL over 5-year follow-up. %TWL at all follow-up points

	Non-banded			Banded			Total
	Median	Mean ± SD	*N*	Median	Mean ± SD	*N*	*P**
%TWL 3 months	15.9	16.0 ± 4.52	141	20.1	20.5 ± 5.30	161	** < 0.001**
%TWL 1 year	30.1	30.4 ± 6.99	150	36.1	35.6 ± 6.53	145	** < 0.001**
%TWL 2 years	31.3	31.4 ± 8.59	137	36.5	35.6 ± 7.91	153	** < 0.001**
%TWL 3 years	27.9	28.6 ± 8.67	103	34.0	34.0 ± 8.47	105	** < 0.001**
%TWL 4 years	28.0	27.4 ± 9.01	80	30.3	31.0 ± 9.57	76	**0.027**
%TWL 5 years	27.7	27.6 ± 9.02	135	32.6	32.6 ± 9.09	161	** < 0.001**

*TWL*, total weight loss. *SD*, standard deviation. **P*-value calculated with Mann–Whitney U test

**Table 3:** EWL over 5-year follow-up. %EWL at all follow-up points

	Non-banded			Banded			Total
	Median	Mean ± SD	N	Median	Mean ± SD	N	P*
%EWL 3 months	37.1	38.2 ± 12.6	141	47.7	49.4 ± 15.8	161	** < 0.001**
%EWL 1 year	70.8	72.5 ± 20.0	150	83.5	85.7 ± 20.2	145	** < 0.001**
%EWL 2 years	74.6	74.5 ± 22.5	137	86.2	85.4 ± 21.2	153	** < 0.001**
%EWL 3 years	67.4	69.0 ± 23.3	103	80.6	82.4 ± 22.7	105	** < 0.001**
%EWL 4 years	69.3	66.2 ± 23.6	80	72.3	73.4 ± 24.5	76	0.10
%EWL 5 years	64.6	65.2 ± 22.1	135	78.2	77.6 ± 23.0	161	** < 0.001**

*EWL*, excess weight loss. *SD*, standard deviation. **P*-value calculated with Mann–Whitney U test

**Table 4:** Comorbidities at 5-year follow-up. Number of patients who reported remission of their comorbidities

	Non-banded	Banded	Missing	Total	*P* value*
Hypertension remission at 5 years (%)	21/54 (38.9)	33/70 (47.1)	26	150	0.37
Diabetes remission at 5 years (%)	24/37 (64.9)	28/41 (68.3)	15	93	0.81
Dyslipidemia remission at 5 years (%)	9/19 (47.4)	5/16 (31.3)	31	71	0.49
OSAS remission at 5 years (%)	11/18 (61.1)	32/46 (69.6)	8	74	0.56

**P*-value calculated with Fisher Exact test. *OSAS*, Obstructive Sleep Apnea Syndrome

**Table 5:** Comorbidities at 5-year follow-up. Number of patients who reported improvement of their comorbidities

	Non-banded	Banded	Missing	Total	*P* value*
Hypertension improved at 5 years (%)	45/54 (83.3)	50/70 (70.4)	26	150	0.14
Diabetes improved at 5 years (%)	32/37 (86.5)	35/41 (85.4)	15	93	1.00
Dyslipidemia improved at 5 years (%)	13/19 (68.4)	8/16 (50)	31	71	0.32
OSAS improved at 5 years (%)	14/18 (77.8)	33/46 (71.7)	8	74	0.76

**P*-value calculated with Fisher Exact test. *OSAS*, Obstructive Sleep Apnea Syndrome
